# Nucleolar organizer region banding in crossbred and non-descript pigs of India

**DOI:** 10.14202/vetworld.2018.1371-1375

**Published:** 2018-10-01

**Authors:** V. Harshini, K. Sakunthala Devi, B. Punya Kumari, J. Suresh

**Affiliations:** Department of Animal Genetics and Breeding, Sri Venkateswara Veterinary University, Tirupati, Andhra Pradesh, India

**Keywords:** silver-stained nucleolar organizer regions, large white Yorkshire crossbred pig, non-descript pig, silver staining

## Abstract

**Aim:**

The objective of this experiment was to study the nucleolar organizer region (NOR)-banding pattern in Large White Yorkshire (LWY) crossbred and non-descript pigs and finding differences in the number of NORs between animals and between genetic groups.

**Materials and Methods:**

The experiment was carried out on 15 females, and 15 males of LWY crossbred and non-descript pigs to study NOR-banding pattern by employing ammoniacal silver staining technique.

**Results:**

A total of 63 and 65 number of good metaphases were prepared in LWY crossbred, and non-descript pigs and a total of 168 and 143 number of NORs were detected on the 8th and 10th chromosomes in both genetic groups, respectively. The mean number of NORs per metaphase was 2.67 and 2.20 in LWY crossbred and non-descript pigs, respectively. LWY crossbred pig had high mean number of silver-stained NORs (Ag-NORs) per metaphase compared to non-descript pig. In general, it was observed that the highest frequency of metaphases (%) examined had two number of NORs, while the lowest frequency (%) had four number of NORs. The number of NORs observed per metaphase on secondary constrictions of the 8th and 10th chromosome pair in both genetic groups ranged from 2 to 4. The Chi-square test of significance revealed that the observed frequencies do not differ significantly from the expected frequencies.

**Conclusion:**

The results confirmed differences across breeds in occurrence and number of NORs on chromosomes in pigs. The mean numbers of NORs present per metaphase vary between the animals indicating the existence of polymorphism for the number of NORs. A higher number of Ag-NORs were observed on chromosome pair 10 in both the genetic groups. It was concluded that NORs were more morphologically distinct and greater on chromosome pair 10 than on pair 8, which suggests a dominant role of chromosome 10 in the global production of ribosomal RNA.

## Introduction

The nucleolar organizer regions (NORs) identified as secondary constrictions in mitotic chromosomes referred as NORs are responsible for the structure, organization, and formation of nucleoli in the process of cell protein synthesis [1], which are then a base for the establishment of ribosomal subunits, which facilitate the synthesis of proteins [2]. NOR bands constitute structural non-histone proteins that are specifically associated with NOR and bind to ammoniacal silver nitrate [3]. For Sus domestica, the NORs occur in the 8^th^ and 10^th^ pair of chromosomes on short arms close to the centromere [4,5]. Structure, number, and morphology of NOR may be specific to populations, species, and subspecies.

Changes in chromosome structure and number can alter the number and structure of NOR. Robertsonian translocations may cause losses of NOR. Species which have limited gene exchange due to geographical isolation, have elevated karyotype and NOR variety [6]. NOR banding can be practiced for chromosomal study with double satellites, chromosome polymorphisms and structural abnormalities involving satellite regions [7,8]. NORs size variants can be applied for studies on differentiation of pig breeds as well as estimation of genetic distance (or) evolutionary relationships in domestic pig (or) between domestic and wild pig [9]. NORs can play a role as candidate markers of parental species [10], chromosome markers in fish cytotaxonomy [11,12].

The objective of this experiment was to study the NOR-banding pattern in Large White Yorkshire (LWY) crossbred and non-descript pigs and finding differences in the number of NORs between animals and between genetic groups.

## Materials and Methods

### Ethical approval

During collection of blood samples from pigs, attention had been paid to minimize pain to the animal and the Institutional Animal Ethics Committee clearance is not required since it involves the only collection of blood samples from experimental animals.

### Experimental animals

The present cytogenetic study was carried out on 15 males and 15 females of LWY crossbred pigs maintained at all India coordinated research project on pig, College of Veterinary Science, Tirupati, Chittoor district of Andhra Pradesh, and on non-descript pigs maintained by the farmers in and around Tirupati.

### Technique

**Figure-1:**
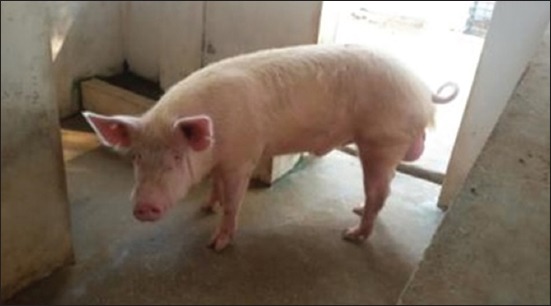
Large white Yorkshire crossbred male maintained in farm conditions (at all India Coordinated Research Project on pig, Tirupati).

**Figure-2:**
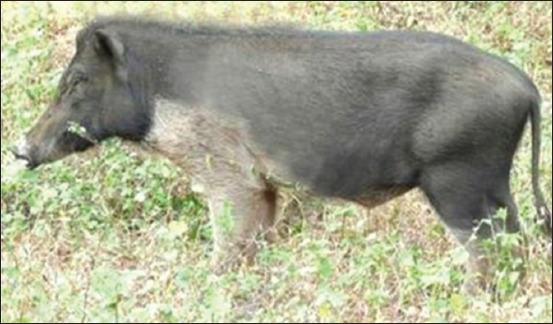
Non-descript male pig in a farmers herd at field conditions.

The photographs of crossbred and non-descript pig included in the present study are shown in Figures-1 and 2. Short-term lymphocyte culture technique, as described by Moorehead *et al*. [13] with slight modifications, was followed for karyotyping and the ammoniacal silver staining procedure as described by Howell and Black [14] was followed with minor modifications for NOR-banding technique. The unstained slides were heated on warmed up to 70°C. About four drops of colloidal developer and eight drops of silver nitrate (50%) were placed on coverslip and slides were kept on the coverslip to enable the chromosomes to take stain for 2 min (or) till the silver stain changed to golden yellow color. The coverslip was removed, and excess stain was washed with deionized water and counterstained with 2% Giemsa for 20 s. The air-dried slides were observed under a bright field microscope.

**Figure-3:**
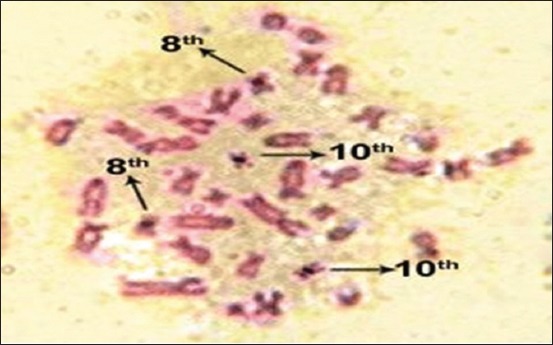
Nucleolar organizer region banding of large white Yorkshire crossbred male pigs.

**Figure-4:**
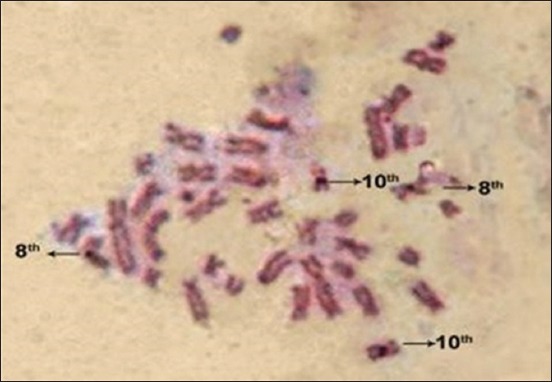
Nucleolar organizer region banding of non-descript male pigs.

The silver-stained NORs (Ag-NORs) were visualized as black spherical bodies on yellow-brown chromosome arms. The good metaphases showing the NORs clearly were photographed and shown in Figures-3 and 4. The number of NORs per metaphase was counted and subjected to the analysis.

### Statistical analysis

The frequency distribution of number of metaphases containing the Ag-NOR bands was tested for significance by Chi-square test [15].

## Results and Discussion

In the present study, a total of 63 and 65 number of good metaphases were examined in LWY crossbred, and non-descript pig and a total of 168 and 143 NORs were detected in both genetic groups, respectively (Table-1). The overall mean number of NORs observed per metaphase was 2.67 and 2.20 in LWY crossbred and non-descript pigs, while [16-18] reported similar values in Dutch Landrace, Yorkshire, Norwegian Landrace, Finish Landrace, Korean native, Xiang, and Min breed of pig. It was observed that LWY crossbred pig had high mean number of Ag-NORs per metaphase (2.67) compared to non-descript pig (2.20) (Table-1). The mean number of NORs present per metaphase in this study varies between the animals indicating the existence of polymorphism for the number of NORs. The highest frequency of metaphases (47.61% in LWY crossbred and 81.54% in non-descript pigs) had two number of NORs, whereas the lowest frequency of metaphases (7.94% in LWY crossbred and 3.07% in non-descript pigs) had four number of NORs (Table-2). It was observed that the frequency of the metaphases reduced as the number of NORs per metaphase increased which was in accordance with the reports of Devi *et al*. [19]. The Chi-square test of significance as revealed that the observed frequencies do not differ significantly from the expected frequencies.

**Table-1:** Number of metaphases examined, number of NORs detected and mean number of NORs per metaphase.

Breed	Number of metaphases examined	Number of NORs detected	Mean number of NORs per metaphase
LWY crossbred pigs	63	168	2.67
Non-descript pigs	65	143	2.20

NORs=Nucleolar organizer region, LWY=Large white Yorkshire

**Table-2:** Frequency distribution of NORs per metaphase in LWY and non-descript pigs.

Number of NORs per metaphase		Frequency (number of metaphases)		Percentage	
		
LWY	ND	LWY	ND	LWY	ND
2	2	30	53	47.61	81.54
3	3	28	10	44.44	15.39
4	4	5	2	7.94	3.07
Total		63	65	100.00	100.00

LWY=Large white Yorkshire, NORs=Nucleolar organizer regions

Prominent NORs were observed on chromosome number 10, suggesting a dominant role of the 10^th^ chromosome in the production of ribosomal RNA, while the NORs of chromosome 8^th^ were varied in staining and showed lesser Ag-NORs. Słota [5] and Mellink *et al*. [20] indicated that the silver deposits on pig chromosomes of pair 10 were greater than those of pair 8 in the individuals analyzed.

In LWY crossbred pig, single silver deposit [21] was observed most frequently on the 8^th^ chromosome pair, whereas only in nine cases two silver deposits occurred on both homologous chromosome pairs. Of total 63 metaphases, 20 number of metaphases had no Ag-NORs on chromosome number 8. On chromosome pair 10, it was observed that two silver deposits were most frequent [22] and 10 metaphases were found with single silver deposits (Table-3).

**Table-3:** Number of metaphases demonstrating the presence of Ag-NORs on chromosomes of LWY crossbred and non-descript pigs.

Breed	Chromosome pair 8 variant			Chromosome pair 10 variant		
	
	+/+	+/−	−/−	+/+	+/−	−/−
LWY crossbred pigs	09	34	20	53	10	−
Non–descript pigs	05	51	09	17	48	−
Total	14	86	27	49	79	−

LWY=Large white Yorkshire, Ag-NORs=Silver-stained nucleolar organizer region

In non-descript pigs, 51 single silver deposits were observed on one homologous pair of chromosome pair 8^th^ and only five cases were shown with two silver deposits on both homologous chromosome and nine number of cases had no silver deposits. On chromosome pair 10, the majority of metaphases showed single silver [23] deposits on one homologous pair, whereas 17 number of cases recorded with single silver deposits on both homologous pairs (Table-3).

The frequency of occurrence of Ag-NORs in both the breeds and the presence (+) or absence (−) of silver deposits on the chromosome pair 8 and 10 is given in Table-4. On the 8^th^ chromosome pair, a total of 52 and 61 number of NORs were observed with mean number of 1.76 and 2.16 regions per animal in LWY crossbred and non-descript pigs. The percentage share of deposits on the 8^th^ pair of homologous accounted for 44.16% and 51.67% in LWY and non-descript pigs, respectively.

**Table-4:** Frequency of occurrence of Ag-NORs in LWY crossbred and non-descript pigs (n=30 animals).

Breed	Chromosome pair 8			Chromosome pair 10		
	
	Number of Ag-NORs observed	Mean number of NORs per animal	Share of deposits (%)	Number of Ag-NORs observed	Mean number of NORs per animal	Share of deposits (%)
LWY crossbred pig	52	1.76	44.16	116	3.87	96.67
Non–descript pig	61	2.16	51.67	82	2.73	68.33

LWY=Large white Yorkshire, Ag-NORs=Silver-stained nucleolar organizer region

A total of 116 and 82 numbers of NORs were noted on the 10^th^ chromosome in LWY crossbred and non-descript pig with mean number of 3.87 and 2.73 regions per animal, respectively (Table-4). The share of silver deposits was calculated against their theoretically possible number, i.e., one individual can have four NORs, and so for 30 number of individuals of a given breed 100% accounted for 120 (30×4) Ag-NORs. The percentage share of deposits on the 10^th^ pair of homologous accounted for 96.67% and 68.33% in LWY and non-descript pigs, respectively.

NORs of chromosome 10 are transcriptionally active in all cells and considered as the main site of rRNA production which was corroborated with the findings of previous studies [1,9,17,20,22,23], whereas the NORs of chromosome 8 were not active to a variable degree. Liu *et al*. [24] stated that the polymorphism of Ag-NORs in pigs is caused mainly by differences in frequency of silver deposits on chromosome 8.

## Conclusion

The results confirmed differences across breeds in occurrence and number of NORs on chromosomes in pigs. The mean number of NORs present per metaphase varies between the animals indicating the existence of polymorphism for the number of NORs. A higher number of Ag-NORs were observed on chromosome pair 10 in both the genetic groups. It was concluded that NORs were more morphologically distinct and greater on chromosome pair 10 than on pair 8, which suggests a dominant role of chromosome 10 in the global production of ribosomal RNA.

## Authors’ Contributions

VH and KSD planned and designed the whole study. VH collected samples, performed technique, and analyzed the data. KSD played a key role in data analysis. KSD, BPK, and JS helped during manuscript writing, crosschecking, and revision. All authors read and approved the final manuscript.
